# Supplementary material for: Content-rich biological network constructed by mining PubMed abstracts
Source: BMC Bioinformatics. 2004 Oct 8;5:147. doi: 10.1186/1471-2105-5-147 (PMC528731; doi:10.1186/1471-2105-5-147)
Supplement: Additional File 2 — The original results of the above study (non-essential files are deleted to keep the file size under the limit set by BMC bioinformatics). [file 1471-2105-5-147-S2.bz2 › chilibotAdditionalFile2/dip05/682ID11866514E17997/html/RPOA_FIS.html]

 


 **RPOA** and **FIS** 
  
Found 1 abstracts in PubMed, retrieved 1.  
 

 What does Google say? 
 PDF only 
| .edu only 

---

- J Bacteriol, 2002   **The RNA polymerase alpha subunit **[RPOA]** from Sinorhizobium meliloti can assemble with RNA polymerase subunits from Escherichia coli and function in basal and activated transcription both in vivo and in vitro.** .
  Sinorhizobium meliloti, a gram negative soil bacterium, forms a nitrogen fixing symbiotic relationship with members of the legume family.
  To facilitate our studies of transcription in S. meliloti, we cloned and characterized the gene for the alpha subunit of RNA polymerase RNAP .
  S. meliloti **rpoA** encodes a 336 amino acid, 37 kDa protein.
  Sequence analysis of the region surrounding **rpoA** identified six open reading frames that are found in the conserved gene order secY SecY adk Adk rpsM S13 rpsK S11 **rpoA** alpha rplQ L17 found in the alpha proteobacteria.
  In vivo, S. meliloti **rpoA** expressed in Escherichia coli complemented a temperature sensitive mutation in E. coli **rpoA**, demonstrating that S. meliloti alpha supports RNAP assembly, sequence specific DNA binding, and interaction with transcriptional activators in the context of E. coli.
  In vitro, we reconstituted RNAP holoenzyme from S. meliloti alpha and E. coli beta, beta , and sigma subunits.
  Similar to E. coli RNAP, the hybrid RNAP supported transcription from an E. coli core promoter and responded to both upstream UP element and **Fis** dependent transcription activation.
  We obtained similar results using purified RNAP from S. meliloti.
  Our results demonstrate that S. meliloti alpha functions are conserved in heterologous host E. coli even though the two alpha subunits are only 51% identical.
  The ability to utilize E. coli as a heterologous system in which to study the regulation of S. meliloti genes could provide an important tool for our understanding and manipulation of these processes.
